# Activity of the human immortalized endothelial progenitor cell line HEPC-CB.1 supporting in vitro angiogenesis

**DOI:** 10.1007/s11033-020-05662-6

**Published:** 2020-07-23

**Authors:** Aneta Kantor, Agnieszka Krawczenko, Aleksandra Bielawska-Pohl, Danuta Duś, Catherine Grillon, Claudine Kieda, Karol Charkiewicz, Maria Paprocka

**Affiliations:** 1 Hirszfeld Institute of Immunology and Experimental Therapy, Polish Academy of Sciences, Rudolfa Weigla 12, 53-114 Wroclaw, Poland; 2 Centre de Biophysique Moléculaire, CNRS UPR 4301, Rue Charles Sadron, 45071 Orléans, France; 3 Medical University of Bialystok, M. Sklodowskiej-Curie 24A, 15-276 Bialystok, Poland

**Keywords:** Angiogenesis, EPC, EPC function, EPC secretome, Regeneration

## Abstract

The human HEPC-CB.1 cell line with many characteristics of endothelial progenitor cells (EPC) was tested for its proangiogenic properties as a potentially therapeutic compound. HEPC-CB.1 cells’ potential to differentiate into endothelial cells was revealed after treating the cells with a mixture of ATRA, cAMP and VEGF, as shown by the reduced expression levels of CD133, CD271 and CD90 antigens, augmentation of CD146 and CD31, and a decrease in cell clonogenicity. The cooperation of HEPC-CB.1 with the endothelial cell line HSkMEC.2 resulted in the formation of a common network. Tube formation was significantly more effective when resulting from HEPC-CB.1 and HSkMEC.2 cell co-culture as compared to a monoculture of each cell line. The exocrine mechanism of HEPC-CB.1 and HSkMEC.2 cross talk by secreted factors was evidenced using the HEPC-CB.1 supernatant to increase the efficacy of HSkMEC.2 tube formation. The proangiogenic factors produced by HEPC-CB.1 were identified using cytokine antibody array. Out of 120 examined factors, the HEPC-CB.1 cell line produced 63, some with known angiogenic activity. As in vivo the angiogenic process occurs at low oxygen tension, it was observed that in hypoxia, the production of defined factors was augmented. The presented results demonstrate that HEPC-CB.1 cells are able to both cooperate and integrate in a newly formed network and produce factors that help the network formation. The results suggest that HEPC-CB.1 cells are indeed endothelial progenitors and may prove to be an effective tool in regenerative medicine.

**Electronic supplementary material:**

The online version of this article (10.1007/s11033-020-05662-6) contains supplementary material, which is available to authorized users.

## Introduction

Although 22 years have passed since the discovery of endothelial progenitor cells (EPC) [1], they have not yet been clearly defined. Therefore, the definition of EPC cells includes both myeloid angiogenic cells of hematopoietic origin, expressing CD45^+^, and endothelial colony forming cells of mesenchymal origin, CD45 negative. The time necessary to form colonies in vitro is also a feature used to distinguish the two cell types. Cells of myeloid origin form in vitro colonies in about 7 days—for that reason they are often named *early outgrowth* EPC [2]. When the biological properties of these cells are being described, production of biologically active agents such as VEGF or IL8 is assigned to them, but there are no data demonstrating their ability to create vessels, so the term “putative EPC” is used [3]. Cells of mesenchymal origin, forming clones in vitro in about 3 weeks, are usually considered to be *late outgrowth* cells, true EPC, capable of homing to sites of damage/inflammation, adhesion to the endothelium and integrating into the vessel wall as well as of differentiation into functional endothelial cells (EC) [4]. It is assumed that both types of cells in vivo are involved in blood vessel formation and repair, but cells of mesenchymal origin actually form vessels and cells of myeloid origin support this process mainly through the production of appropriate growth factors. Each of them has a different origin and—as many researchers emphasize—functional features. In the Timmermans review about 20 phenotypes of human EPC cells used by different researchers were described [4, 5]. Different combinations of CD34, CD133, CD31, VE-cadherin, CD146, and VEGFR2 markers were applied to discriminate EPC from other cells as to date no EPC specific marker has been found. The lack of a specific marker of EPC cells and very low number of these cells in the organs and circulation cause many problems in identification, isolation and especially application. Only recently have there appeared works attempting to introduce the correct EPC nomenclature [6].

As initial results from animal studies suggested that EPC could bring clinical improvement in patients not eligible for revascularization surgery, experimental therapies, based on the angiogenic potential of EPC, were applied in clinical practice [7, 8]. Currently, about 20 trials are registered at the website ClinicalTrials.gov, where EPC cells are applied to the patients to obtain therapeutic effects. In the clinical trials, distinct populations of cells were used, both unselected and expressing a characteristic marker, often CD34 [8] or CD133 [9–11]. However, selection based on the expression of a single marker is not sufficient to distinguish EPC from other cell types, while isolation based on simultaneous expression of a larger number of markers, e.g. CD31, CD34 and VEGFR2, dramatically reduced the number of obtained cells. Therefore, the main problem in the potential clinical use of EPC appeared to be the limited availability of these cells. One to several hundred million cells [12] isolated from 12 L of blood would give a sufficient number of EPC for clinical application [13]. Therefore, to achieve a sufficient cell number, their multiplication in an ex vivo system is performed in the presence of cytokines and growth factors [14–16]. Another approach is induction of EPC in the circulation by prior injection of growth factors, e.g. G-CSF [17, 18], or isolation of cells from two or more donors. Another possibility to provide a sufficient number of progenitor cells with a well-defined cell type, for basic research and possible clinical use, is their immortalization [19, 20].

A few years ago, our team obtained and described two similar human cell lines that meet several features of EPC [19]. These cell lines, derived from umbilical cord blood, named HEPC-CB.1 and HEPC-CB.2, both express CD133, CD271, CD146, CD90 on their surface but do not express CD45, CD34 or VE-cadherin. Additionally they are able to create capillary-like structures on Matrigel and produce some growth factors critical for endothelial cell viability (e.g. VEGF and IL-8). We postulate that for research purposes a well-defined cell line such as HEPC-CB.1 may be better than heterogeneous mixtures of cells separated from different donors or obtained as a result of in vitro culture.

Our hypothesis is that the HEPC-CB.1 cell line with its specific features and phenotype of EPC at an early stage of development also has potential to become a therapeutic tool. As this work describes, HEPC-CB.1 cells are real EPC with angiogenic properties. Those properties include influencing other endothelial cells directly or indirectly through the biologically active factors they produce, recognizing, connecting and interacting with mature endothelial cells, and differentiating into endothelial cells. Such cells with the features described above could be isolated from the patient, ex vivo immortalized, propagated and then administered to the patient. We believe that the patient's own cells, with HEPC-CB.1 phenotype, could be used in regenerative medicine.

## Materials and methods

### Cells

Human endothelial progenitor cell lines originated from cord blood (HEPC-CB.1) (C. Kieda, Centre National de la Recherche Scientifique, France, European patent no. 1170 3915.6, the USA extended patent no. is 13/521 715) [19] and human normal skin microvascular endothelial cells (HSkMEC.2) (C. Kieda, Centre National de la Recherche Scientifique, France, patent 99–16169) were established according to a method described previously [21]. All endothelial cells were cultured in Opti-MEM GlutaMAX-I medium supplemented with 3% Fetal Bovine Serum (FBS, HyClone, UK) and 1% Penicillin–Streptomycin (Sigma Aldrich, USA) and were routinely passaged using 0.05% trypsin/0.02% EDTA (w/v) solution (IITD PAN, Poland).

Human umbilical vein endothelial cells (HUVEC), isolated from umbilical vein (macrovasculature) and immortalized with hTERT (using a previously described protocol [22]) were cultivated in 199 medium (Lonza, USA) supplemented with 10% FBS (HyClone, UK), l-glutamine (Sigma Aldrich, USA), 1% Penicillin–Streptomycin (Sigma Aldrich, USA) and 200 μg/ml Endothelial Cell Growth Supplement (ECGS, Becton Dickinson, USA.

### Proliferation activity

In order to determine the proliferation properties of studied cells, sulforhodamine B tests were performed. The cells were detached with a trypsin/EDTA solution, centrifuged and suspended in 100 μl of OptiMEM medium with 1% FCS at 2 × 10^4^ cells per well of a flat-bottom 96-well culture plate. Cell cultures were carried out for 24, 48, 72 and 96 h under normoxia or hypoxia conditions. Cultures were discontinued by fixing the cells for 60 min at 4 °C cold 50% trichloroacetic acid (TCA). Plates with fixed cells were washed five times with distilled water and dried. Then 50 μl of a 0.4% solution of sulforhodamine B (SRB) in 1% acetic acid was added to the wells and incubated in the dark for 30 min at room temperature. Unbound SRB was removed by washing the wells five times with 1% acetic acid. After re-drying, 150 μl of a 10 mM Tris solution was added to the wells and shaken for 30 min to dissolve the SRB. The absorbance at 570 nm was measured using a Victor^2^ 1420 Multilabel Counter Wallac plate reader.

### Differentiation of HEPC-CB.1 cells

For the differentiation of HEPC-CB.1 cells, cells were cultured in OptiMEM GlutaMAX-I complete medium (medium with 1% FCS and 1% Penicillin–Streptomycin). Twice a week, all trans retinoic acid (ATRA, 1 μM solution, Sigma-Aldrich, USA), cyclic adenosine monophosphate (cAMP, 0.5 mM solution, Sigma-Aldrich, USA) and vascular endothelial growth factor (VEGF, 50 ng/ml, R&D Systems, USA) were added to the culture. The cells treated in this way are referred to as “HEPC-CB.1 differentiated” cells. Control cells were cultured in OptiMEM GlutaMAX-I complete medium and are referred to as “HEPC-CB.1 control”.

### Immunostaining of HEPC-CB.1 cells

Phenotype of cultured cells was analyzed using directly labeled monoclonal mouse antibodies: PE-conjugated anti-CD133 and FITC-conjugated anti-CD271 (Miltenyi Biotec, Germany); PE-conjugated anti-CD90 (BD Biosciences, USA); PE-conjugated anti-CD105, PE-conjugated anti-HLA-DR (BD Pharmingen) and anti-CD146 (R&D Systems, USA). All antibodies were used at the concentration of 1 to 5 µl of antibody for 1 × 10^5^ cells in 50 µl of PBS supplemented with 1% FBS, as suggested by the manufacturer, and incubated for 30 min at 4 °C. After incubation cells were carefully washed. IgG isotype matched, PE or FITC labeled immunoglobulins were used as controls. Cells were analyzed by flow cytometry using FACSCalibur (Becton Dickinson, CA). Data were presented using WinMDI 2.7 software.

### Evaluation of expression of CD31 mRNA by real-time RT-PCR

The culture of HEPC-CB.1 cells was performed in OptiMEM GlutaMAX-I complete medium as a control, and in the test group in OptiMEM GlutaMAX-I complete medium with addition of a mixture of ATRA + cAMP + VEGF. Total cellular RNA was isolated from 4 × 10^6^ cells using a NucleoSpin RNA kit (MACHEREY–NAGEL, Germany). First strand cDNA synthesis was performed by reverse transcription of 1 μg of total RNA using the RevertAid First Strand cDNA Synthesis Kit for RT-PCR (Thermo Scientific, USA). Real-time qPCR amplification and analysis were performed using the ViiA 7 Real-Time PCR System (Thermo Fisher Scientific, USA). The qPCR assay was performed with Kapa Probe Fast ROX Low qPCR MasterMix and the TaqMan probe Hs00169777_m1 PECAM1. All samples, the internal control (GAPDH), and the non-template control (water to confirm the absence of DNA contamination in the reaction mixture) were performed in triplicate. Relative expression level of the studied gene was evaluated with the comparative method (ΔΔCt).

### Formation of colonies

The EPC colony-forming capacity was evaluated similarly to the classical Hill’s method. HEPC-CB.1 cells were grown in OptiMEM GlutaMAX-I complete medium. Increasing concentrations of cells (from 7 to 250 cells/ml/well) were seeded into 12-well plates. Fibrin coating was not applied as cells are able to adhere to plastic easily. After 4, 6 and 8 days of culture clones containing over 10 cells were counted under an Olympus CK30 inverted microscope. The presented results and images were obtained using an Olympus CKX 41 microscope using the Stream Start program.

### Preparation of supernatant from HEPC-CB.1 cell line

To prepare the supernatant, 7.5 × 10^5^ cells in 1.2 ml of OptiMEM complete medium/well were plated on a 6-well plate and cultured in standard conditions. After 5 h, when cells adhered to the culture vessel and formed a confluent layer, the medium was changed and cells were cultured under normoxic (20% O_2_) or hypoxic (1% O_2_) conditions for 24 h. Then, the supernatant was collected and centrifuged (5 min, 20 °C, 500 g) to remove cells. The liquid was transferred to cryotubes and frozen by quickly dipping tubes in liquid nitrogen and then transferred to − 80 °C.

### Capillary-like structures formation assay

Matrigel matrix, both complete and with reduced growth factors (BD Biosciences), was diluted (1:1) in OptiMEM medium at 4 °C, distributed in 96-well microplates in a volume of 40 µl and allowed to polymerize at 37 °C for 30 min.

If necessary, for fluorescent cell staining, 1 × 10^6^ cells were resuspended in 1 ml of OptiMEM medium. Next 6 μl of staining reagent Vybrant DiO or Vybrant DiD was added and the mixture was incubated at 37 °C. After 15 min FCS was added for a final concentration of 2% to terminate the reaction and cells were washed with medium.

HSkMEC.2 and HEPC-CB.1 (1.5 × 10^4^ cells for monoculture or 0.75 × 10^4^ cells of each type in the case of co-culture) were seeded on Matrigel-coated microplates in 100 µL of the appropriate medium:

- OptiMEM GlutaMAX-I medium with 1% FCS
- 50% supernatant: supernatant from HEPC-CB.1 cells, diluted 1:1 with OptiMEM GlutaMAX-I medium containing 1% FCS
- 100% supernatant: undiluted supernatant from HEPC-CB.1 cells

Experiments were performed either in normoxic (20% O_2_) or hypoxic (1% O_2_) conditions.

The direct real-time visualization of the capillary-like structures’ formation was monitored for 24 h using either visible light or fluorescence. The images were obtained using an inverted fluorescence microscope (Axiovert 200 M; Zeiss, Le Pecq, France) equipped with an Axiocam high resolution numeric camera linked to a computer driving the acquisition software AxioVision (Zeiss). As the parameters usually used to evaluate the network do not describe well the differences between different cell lines, angiogenesis was quantified by the number of nodes, by the measurement of capillary-like structures’ total length and by the determination of the mean mesh size, using ImageJ software (NIH, Wayne Rasband, USA).

### Determination of cytokine profile of cells using cytokine antibody array

The secretory profile of the HEPC-CB.1 cell line was evaluated using RayBio C-Series Human Cytokine Antibody Array C1000 and RayBio Custom C-Series Human Cytokine Antibody Array according to the manufacturer’s instructions. Unless otherwise stated, all incubations were performed in the incubation chamber provided by the manufacturer under gentle rotation at room temperature. Protein membranes were placed in incubation chambers and incubated with blocking buffer. After 30 min blocking buffer was removed and 1.2 ml of supernatant from HEPC-CB.1 control or HEPC-CB.1 differentiated cells was added and incubated for 16 h at 4 °C. After this time, the membranes were washed in washing buffers. Then membranes were incubated for 2 h with a biotinylated antibody cocktail and after incubation washed in washing buffers, then incubated for 2 h with HRP-connected streptavidin and washed again.

The reaction was then visualized using a chemiluminescent method in the darkroom. Arrays were placed on a rigid flat surface and incubated for 2 min with a mixture of detection buffer C and detection buffer D. Excess fluid was drained, then a transparent film was applied to the matrix and placed in a light-tight Kodak X-Omat AR photographic film cassette for about 40 s on XAR-5 film, which was next developed using Kodak reagents. Supplementary material Fig. A presents a map of the custom antibody array.

Images were analyzed densitometrically using ImageJ software. Obtained numerical values were entered into Microsoft Excel-based Analysis Software Tools. The results were expressed as a percentage of expression, where the positive control was set to 100% and the negative control to 0% (relative expression, both controls included on the membrane). The cut-off line was set to 5%; all results above 5% were considered as real expression.

### Statistical analysis

All values were presented as mean ± SD values calculated from at least three independent experiments, each consisting of technical duplicates. Results were analyzed through the unpaired t-test using Microsoft Excel software. Results with p < 0.05 were considered statistically significant.

## Results

### Phenotype of HEPC-CB.1 cell line

**Fig. 1:**
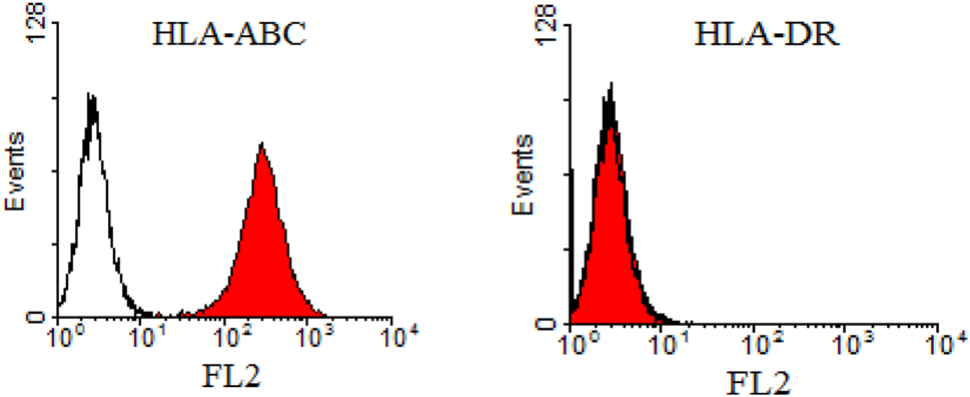
MHC antigen expression on HEPC-CB.1 cells determined by flow cytometry methods. Data are presented as histogram overlays using WinMDI 2.7 software. Empty histograms represent isotypic controls and colored histograms the cell labeled with HLA-ABC or HLA-DR antibodies. Values on the X axis indicate fluorescence intensity (FL2) expressed on the log scale and the number of events is expressed on the Y linear scale

It was demonstrated that the HEPC-CB.1 cell line, obtained from a fraction of human mononuclear cord blood cells, display HLA-ABC antigen, whereas they do not express HLA-DR, which indicates that they may correspond to early progenitors (Fig. 1). These cells expresses a panel of stem cells markers: CD133, CD271 and CD90. This cell line is also positive for markers of progenitors and differentiated endothelial cells: CXCR4, CD44, CD15s, UEA-1 and Dil-Ac-LDL. Moreover, HEPC-CB.1 cells express the endothelial cell markers CD202b, VEGFR2, CD146, CD105 and CD143 and are negative for CD34, CD117, CD38 and CD45. The exact phenotypic characterization of HEPC-CB.1 cells was presented in a previous publication [19].

Because the phenotype of the HEPC-CB.1 cells indicates that they correspond to real EPC, we examined whether they retain the ability to differentiate towards mature endothelium.

### Differentiation of HEPC-CB.1 cells

**Fig. 2:**
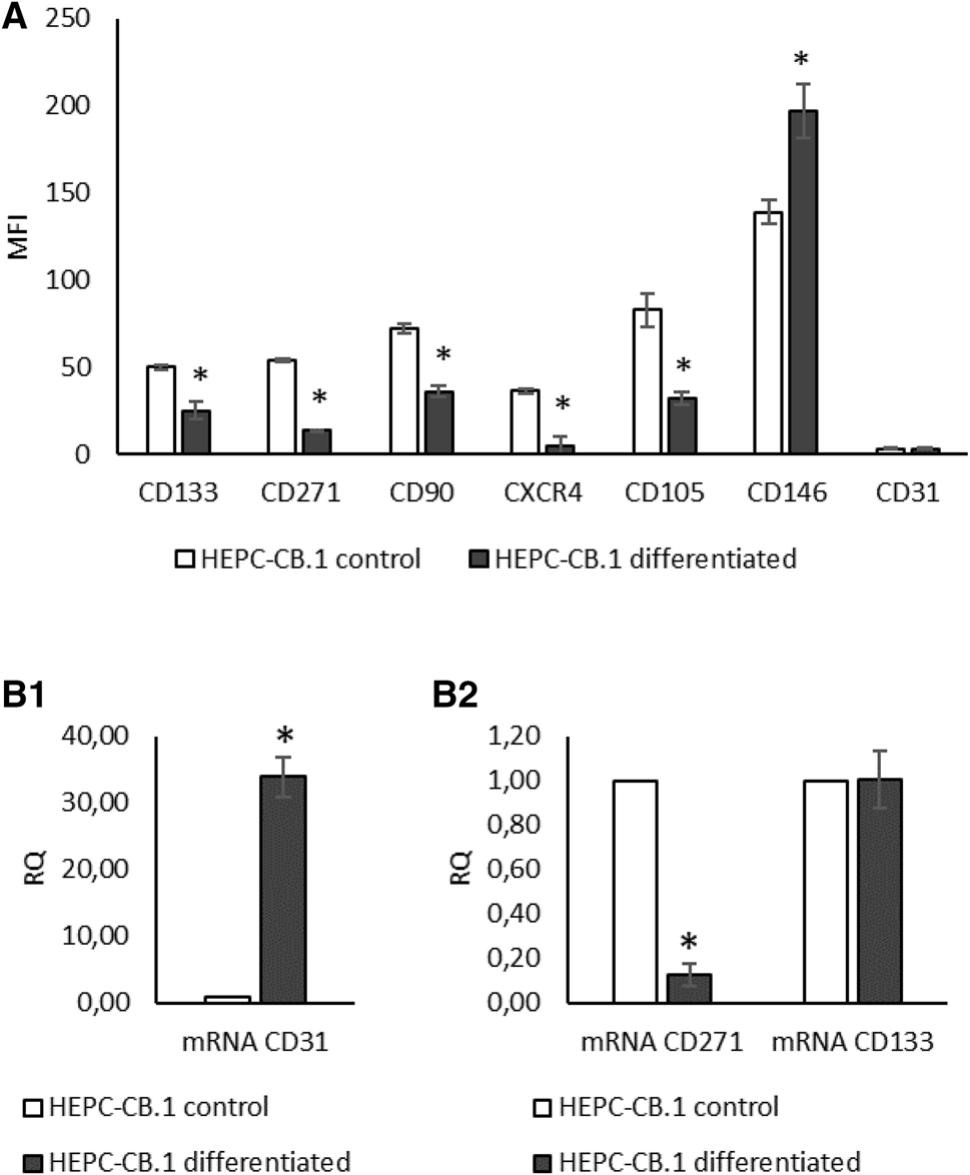
HEPC-CB.1 differentiation by ATRA, cAMP and VEGF mixture reduces the level of molecules characteristic for stem cells and upregulates the level of endothelial-specific molecules. **A** Changes in antigen expression determined by flow cytometry methods. The results were presented as mean fluorescence intensity (MFI) ± SD of the representative experiment, determined on the basis of three replicates. **B1**, **B2** Change in the mRNA level evaluated by real-time PCR. The results are presented as fold changes of values obtained for differentiated versus control cells. Relative quantification (RQ) was determined by the 2^−ΔΔCt^ method. The reference gene was GAPDH. *Indicates statistically significant differences (p < 0.05) of differentiated cells (black column) versus control cells (empty column)

A number of attempts to differentiate the HEPC-CB.1 line have been made. Among many factors tested (e.g. VEGF, thymosin β4, ATRA, cAMP, human platelets—alone or in combination), changes in antigen expression levels were observed only when cells were cultured with a mixture of ATRA, cAMP and VEGF. After 3 weeks of culture, a combination of these factors caused partial differentiation, manifested in lowering expression levels of CD133, CD271, CD90, CXCR4 and CD105 antigens and augmentation of CD146 (as evaluated by FACS analysis, at the protein level, Fig. 2A). Increased expression of the endothelial marker CD31 was clearly visible at the mRNA (Fig. 2B1) but not at the protein level (Fig. 2A). The decrease in the level of CD271 protein correlated with the decrease in mRNA level (Fig. 2B2). Such a correlation was not observed for the protein CD133 (Fig. 2A).

Since the differentiation process influenced the antigen expression pattern, we examined whether it also affects another characteristic feature, i.e. the efficiency of colony formation or proliferation rate.

### Proliferation activity

Comparison between the HEPC-CB.1 cell proliferation with mature HSkMEC.2 and HUVEC endothelial cells showed that the cells of both mature endothelial lines proliferate more slowly than progenitor HEPC-CB.1 cells (Supplementary Fig. B). A statistically significant decrease in the proliferation rate of EC lines relative to HEPC-CB.1 cells was observed from 48 h of assay.

The effect of differentiation by mixture of ATRA + cAMP + VEGF on the proliferative capacity of HEPC-CB.1 cells was also determined. HEPC-CB.1 cells after treatment by mixture of ATRA + cAMP + VEGF proliferate more slowly than control cells: statistically significant decreases in the proliferation rate of differentiated versus control cells were observed at 48 h and 96 h of the test. At 96 h of the test, inhibition of the cell proliferation rate of differentiated versus control cells was about 40% (Supplementary Fig. C).

### Ability of HEPC-CB.1 cells to form colonies

**Fig. 3:**
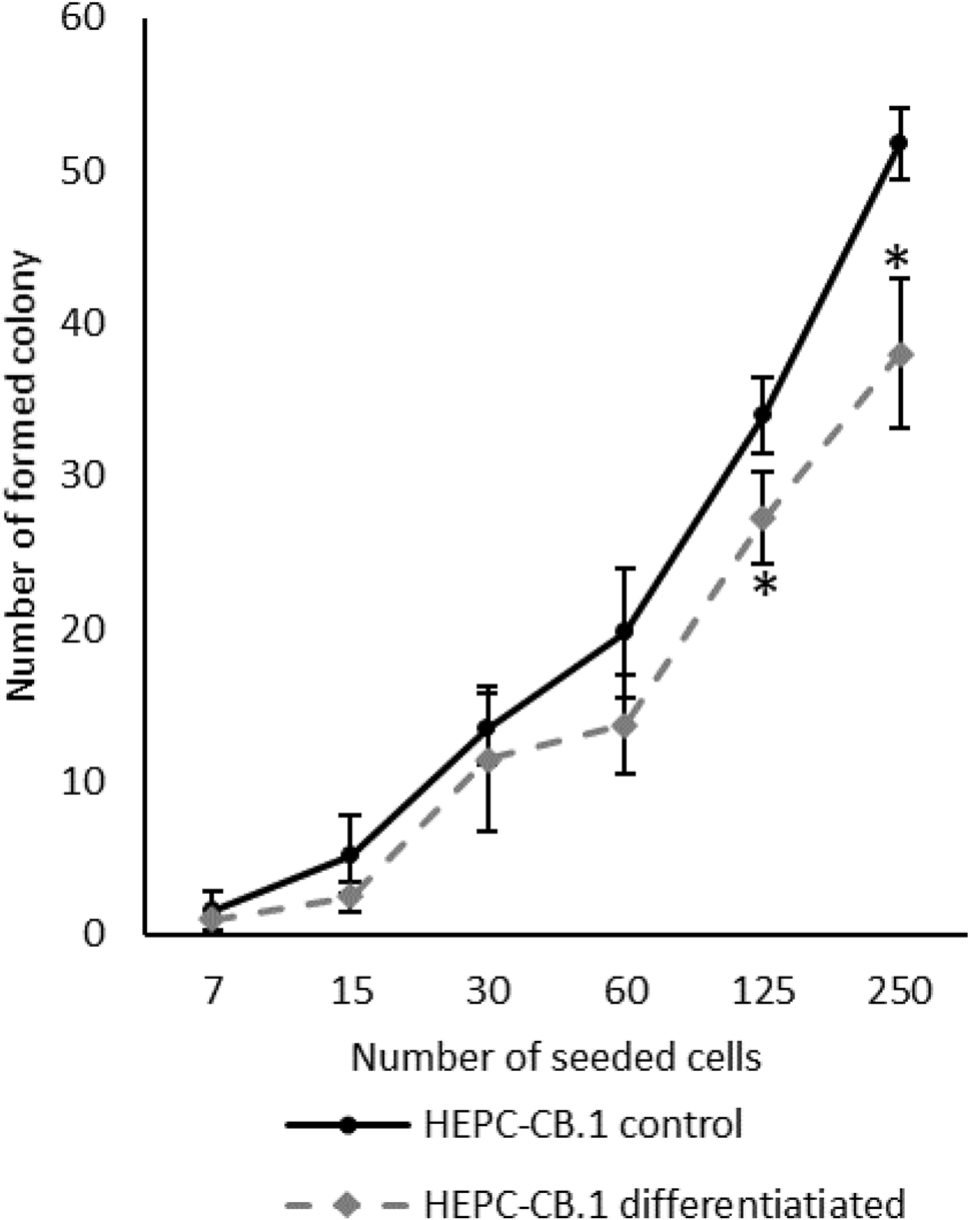
Differentiation process leads to decrease in the cell’s potential for colony formation. Analysis of results on 6th day of experiment. The values in the graphs represent the number of colonies ± SD of the representative experiment, determined on the basis of four independent measurements. P values were determined by t-test. *Indicates statistically significant differences (p < 0.05) between differentiated versus control HEPC-CB.1 cells

One of the effects of the differentiation process is the decrease of the ability of cells to form colonies. As shown in Fig. 3, differentiated HEPC-CB.1 cells formed colonies less efficiently than control cells. Statistically significant differences were demonstrated between control and differentiated HEPC-CB.1 cells in the concentrations 125 and 250 cells/well. Differentiated cells formed about 20 and 27% less colonies, respectively (Fig. 3). Furthermore, colonies created by differentiated cells formed less compact structures (Supplementary Fig. D).

Since the formation of capillary-like structures on the reconstituted extracellular matrix is considered to be characteristic for endothelial cells, we determined the ability of HEPC-CB.1 to create capillary-like structures.

### Angiogenic potential of HEPC-CB.1 cells

**Fig. 4:**
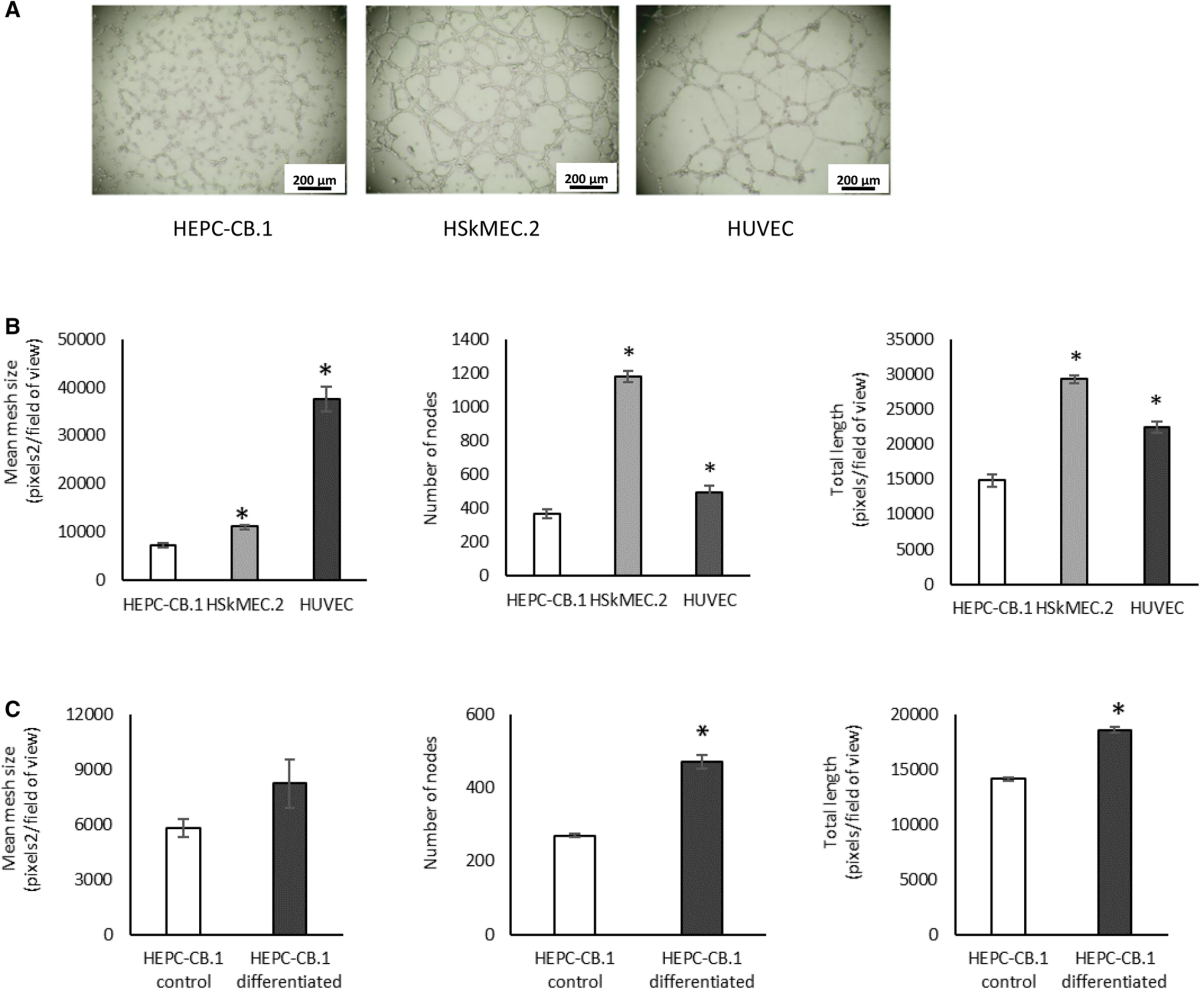
Capillary-like structure formation in Matrigel assay by HEPC-CB.1, HSkMEC.2 and HUVEC cells. A. The images show the tube formation by HEPC-CB.1 as compared to HSkMEC.2 and HUVEC cell lines, magnification ×40. B. Analysis of the results shown in A. C. Differentiation of HEPC-CB.1 cells leads to an increase in the efficiency of capillary-like structure formation. The results were obtained after 6 h of the test using the microscope Axiovert 200 M and AxioVision and ImageJ software. The values in the graphs represent the mean mesh size, number of nodes and total length ± SD of the representative experiment, determined on the basis of three independent measurements. P values were determined by t-test. *Indicates statistically significant differences (p < 0.05): in B. between HSkMEC.2 and HUVEC as compared to HEPC-CB.1 cells and in C. between differentiated and control HEPC-CB.1 cells

HEPC-CB.1 cells were assessed for their angiogenic potential in Matrigel assay. HEPC-CB.1 cells form a capillary-like network on Matrigel matrix. However, as shown in the images, the network is different from those formed by endothelial cell lines: HSkMEC.2, a microvascular endothelial cell line from the skin, and HUVEC, a macrovascular endothelial cell line from umbilical vein (Fig. 4A). When the network formed by HEPC-CB.1 is compared to those formed by mature endothelial cells, it can be seen that the EPC network is not well organized: cells are not always connected and remain more rounded and less stretched. The meshes of their nets are also characterized by a smaller diameter.

If we compare different types of cells (HEPC-CB.1, HSkMEC.2 and HUVEC), forming different types of networks, the best parameter is mean mesh size. Cells with a high value of mean mesh size form large meshes with higher branch length, whereas cells with a low mean mesh size value form a network consisting of a high number of small meshes with a shorter branch length.

Calculations have shown that out of the three lines compared, the network with the largest meshes was formed by HUVEC cells. The meshes of their networks were 3–5 times larger than those of other lines studied. Statistically significant differences were also found between HEPC-CB.1 and HSkMEC.2 cells—meshes formed by HSkMEC.2 cells were 55% bigger than those formed by HEPC-CB.1 cells. However, the number of nodes and the total length of vessels were the highest for HSkMEC.2 cells, followed by HUVEC and HEPC-CB.1 (Fig. 4B). We also found that the differentiation process increased HEPC-CB.1 cell potential to create capillary-like structures. All three analyzed parameters were higher in differentiated cells: they formed 70% more nodes, nets with larger mesh size and greater total length (Fig. 4C).

**Fig. 5:**
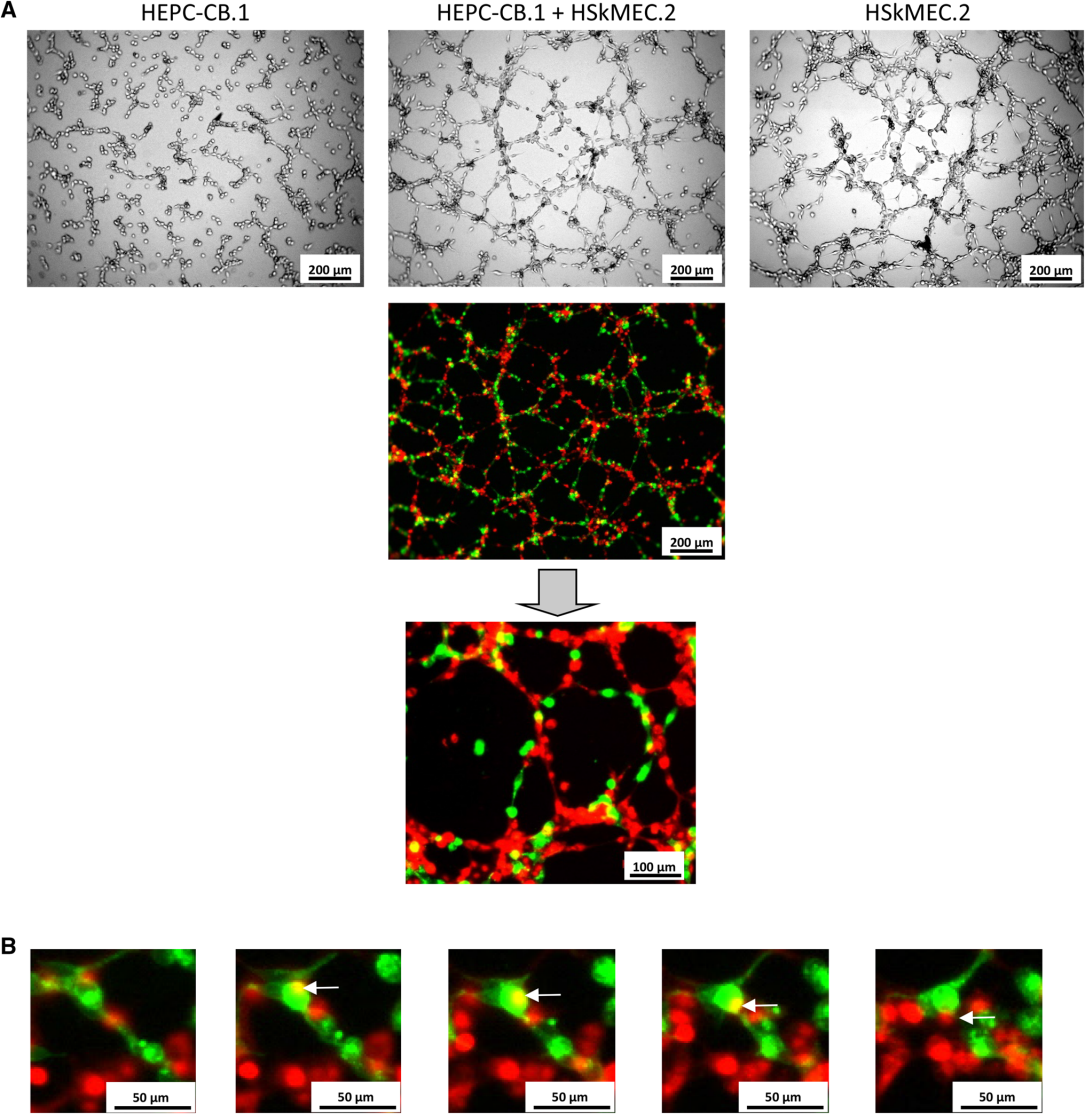
Cooperation of HEPC-CB.1 and HSkMEC.2 cells in the capillary-like structure formation on Matrigel. HEPC-CB.1 cells were stained with Vybrant DiO (green), HSkMEC.2 cells were stained using Vybrant DiD (red). **A** Capillary-like structures formed in the co-culture consist of alternating HEPC-CB.1 and HSkMEC.2 cells. Images taken after 18 h of culture. **B** Co-culture of green stained HEPC-CB.1 cells with red stained HSkMEC.2 cells. Frame-by-frame images taken every hour are presented. Visible yellow spots correspond to the overlap of colors, indicating cell movement in two different planes. Images were taken using the microscope Axiovert 200 M

Incorporation into the existing vascular network is a widely recognized ability of EPC cells in vivo. Capillary-like structures formed by the mixture of HEPC-CB.1 and HSkMEC.2 cells differ from those formed by HEPC-CB.1 and HSkMEC.2 cultured alone as evaluated by standard microscopy. Cells in co-culture formed a network with higher mean mesh size and number of nodes, as presented in Fig. 5A. Using fluorescence microscopy we demonstrated that HEPC-CB.1 cells are able to build in the network formed by mature endothelium. We also observed that the individual capillary-like structures formed in the co-culture consist indeed of alternately arranged precursors and mature cells. Observing images of the capillary-like structure formation by a mixture of both cells labeled with red and green dyes, yellow spots are sometimes found. Following frame-by-frame image analysis, taken every hour, we observed that yellow is just an overlap of colors, indicating cell movement during the angiogenic test (Fig. 5B).

**Fig. 6:**
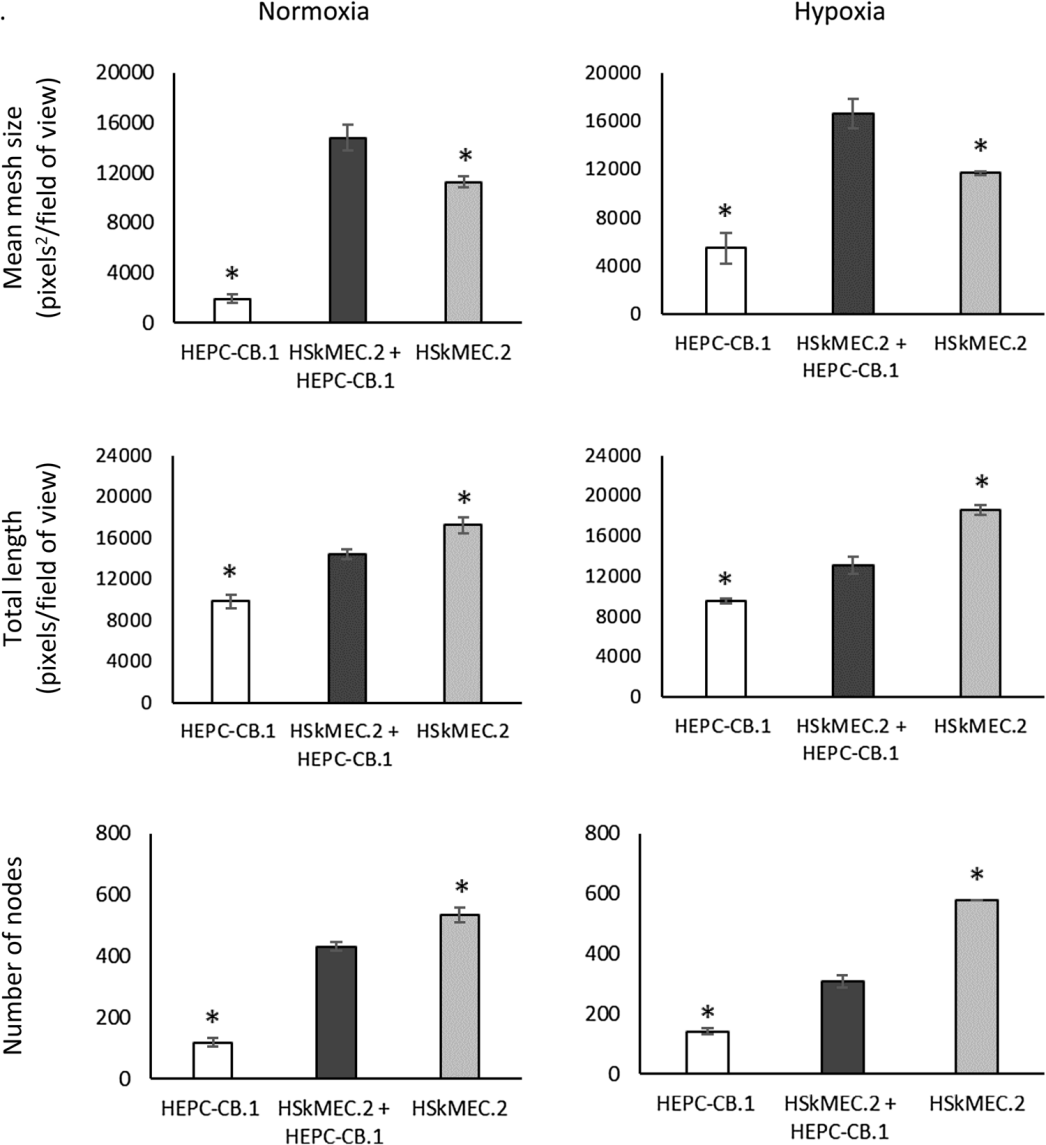
Co-culture of HEPC-CB.1 and HSkMEC.2 cells on Matrigel matrix leads to an increase in the mean mesh size compared to the monoculture of these cells, under both normoxic and hypoxic conditions. Analysis of the images using AxioVision and ImageJ software. The values in the graphs represent the mean mesh size, number of nodes and total length ± SD of the representative experiment, determined on the basis of three independent measurements. P values were determined by t-test. *Indicates statistically significant differences (p < 0.05) between HEPC-CB.1 or HSkMEC.2 cells against co-culture of both cell lines

To evaluate precisely the efficiency of capillary-like structure formation by co-cultures of HEPC-CB.1 and HSkMEC.2 cells as compared to monocultures of these cells, as well as to better imitate the real angiogenesis conditions inside the organs and in the place of damage, the experiments were conducted in normoxic and hypoxic conditions (Fig. 6). We observed a synergistic effect of co-culture of both types of cells on the efficiency of network formation: after 18 h of culture in normoxia, mean mesh size of co-cultures of HEPC-CB.1 with HSkMEC.2 cells was almost 8 times greater than the mean mesh size formed by HEPC-CB.1 cells and 24% larger than the mean mesh size created by HSkMEC.2 alone. In hypoxia, the mean mesh size for co-culture cells was 3 times greater compared to HEPC-CB.1 and about 30% when HSkMEC.2 cells were compared.

When capillary-like structure formation was evaluated by total length and number of nodes, the results obtained for co-cultures were an average of results obtained for cells of both lines cultured separately. The number of nodes and the total length of the network formed by HEPC-CB.1 and HSkMEC.2 cells were higher than those obtained in the HEPC-CB.1 cell monoculture, but smaller than in the monoculture of HSkMEC.2 cells. Statistically significant differences were obtained under both normoxic and hypoxic conditions.

We could also observe that the reduced oxygen concentration caused network creation by HEPC-CB.1 cells with a larger mean mesh size. Under hypoxic conditions, cells formed networks with almost 2.5 times larger meshes*. *This result was not observed for HSkMEC.2 cells.

The observed synergistic effect in the formation of vascular structures by co-cultures seems to be very important when considering cells of a given type as a convenient tool in regenerative medicine. As HEPC-CB.1 are able to stimulate HSkMEC.2 angiogenesis directly in co-culture, it is also interesting to decipher the mechanisms. Is only the direct cell–cell contact responsible for this, or are the factors produced by cells also responsible?

### Effect of factors produced by HEPC-CB.1 cells on angiogenesis

**Fig. 7:**
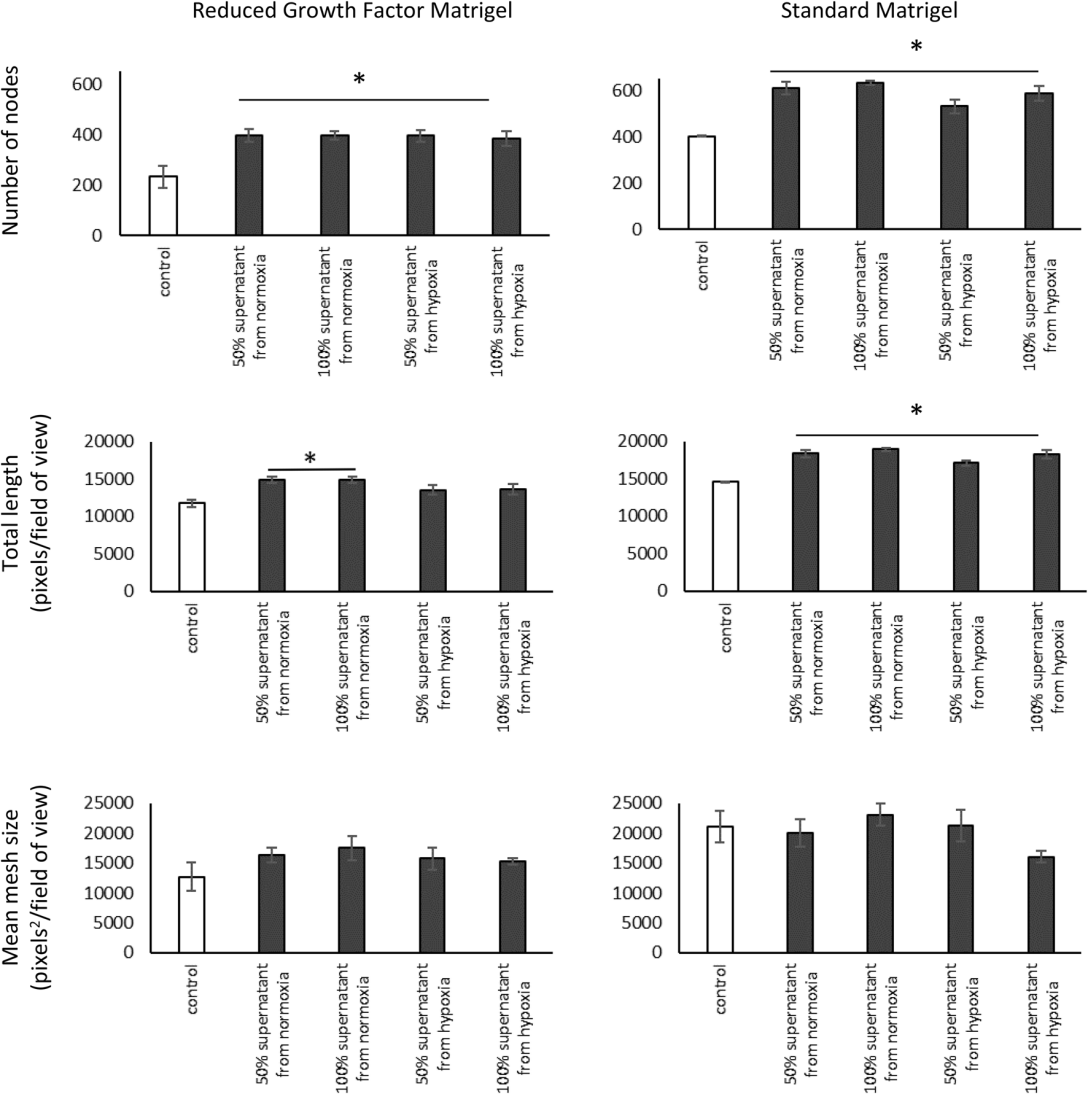
Effect of factors secreted by HEPC-CB.1 cells on the efficiency of capillary-like structure formation by HSkMEC.2 cells on standard Matrigel and on reduced growth factor Matrigel. Analysis using AxioVision and ImageJ software. The values in the graphs represent the mean mesh size, number of nodes and total length ± SD of the representative experiment, determined on the basis of three replicate independent measurements. P values were determined by t-test. * Indicates statistically significant differences (p < 0.05) determined for cells in supernatants (black column) relative to control cells (empty column). 50% supernatant: supernatant from HEPC-CB.1 cells, diluted 1:1 with OptiMEM GlutaMAX-I medium containing 1% FCS. 100% supernatant: undiluted supernatant from HEPC-CB.1 cells

To investigate whether factors produced by HEPC-CB.1 cells can modulate capillary-like structure formation by endothelial cells, cultures of HSkMEC.2 cells with supernatants from HEPC-CB.1 cells, from both normoxic and hypoxic conditions, were performed on standard Matrigel and on reduced growth factor Matrigel. The number of nodes was found to be significantly higher in the presence of EPC cell supernatants. In this case, the number of nodes is the parameter that best reflects the observed differences (Fig. 7).

When Matrigel with a reduced amount of growth factors was used, a statistically significant increase in the efficiency of capillary-like structure formation was demonstrated for the number of nodes and the total length parameters when supernatants from normoxia were used. For hypoxic supernatants only the number of nodes was significantly increased. Cells in supernatants from normoxia formed 70% more nodes (for both 50% and 100% supernatants) and capillary-like structures were longer by about 27% and 26% (for 50% and 100% supernatants, respectively). For supernatants from hypoxia, the number of nodes was 69% higher for cells in 50% supernatant and 64% higher for cells in 100% supernatant.

Similar results were obtained when Matrigel with a standard amount of growth factors was used. A statistically significant increase in the efficiency of capillary-like structure formation was demonstrated as the node number and the total tube length. The number of nodes was 51% higher for cells in 50% supernatants from normoxia, 57% higher for cells in 100% supernatants from normoxia, 32% higher for cells in 50% supernatants from hypoxia and 45% higher for cells in 100% supernatants from hypoxia. Statistically significantly longer capillary-like structures were created by cells in all analyzed supernatants compared to the control.

A larger relative increase in the efficiency of capillary-like structure formation was observed when Reduced Growth Factor Matrigel was used. This matrix contains fewer endogenous growth factors favoring the capillary-like structure formation than the standard Matrigel, thus supporting angiogenesis to a lesser extent, and the influence of secreted factors is more visible.

Since we have shown that cell-free supernatants from HEPC-CB.1 cells exert a proangiogenic effect on the nets formed by HSKMEC.2 cells, an important question arises: what specific pro-angiogenic factors are secreted by these cells? This question seems particularly important if the real EPC cells at this early stage of differentiation are considered as a hypothetical tool in regenerative medicine.

### Secretory properties of HEPC-CB.1 cells

**Fig. 8:**
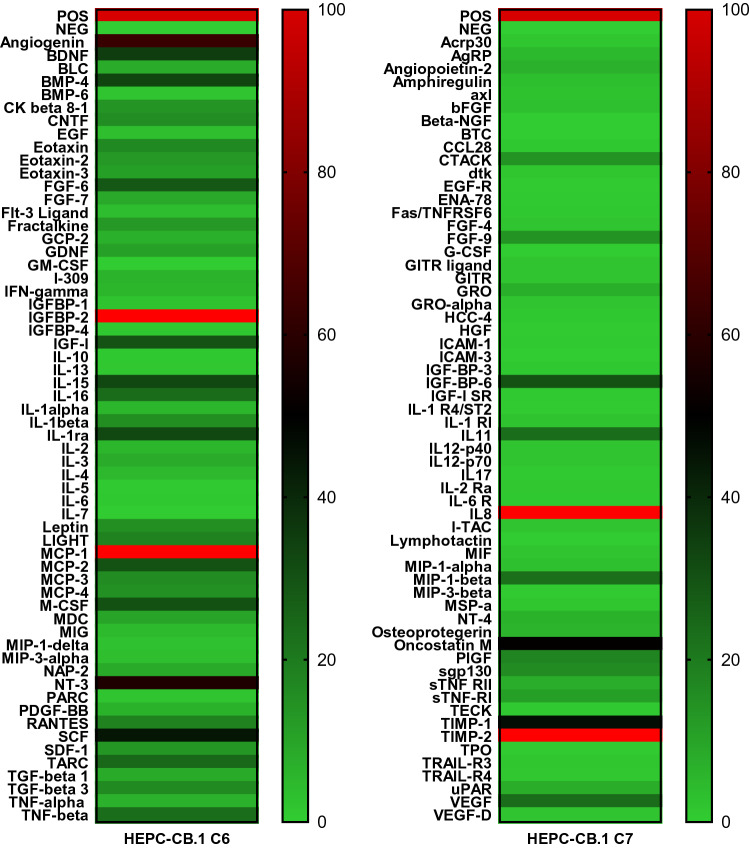
Screening of factors produced by HEPC-CB.1 cells. Supernatants from the HEPC-CB.1 cell line were collected after 24 h of culture in normoxia (20% O_2_). Cytokine secretion was evaluated with RayBio C-Series Human Cytokine Antibody Array C1000. The figure presents a heatmap of tested cytokines. At the top, dark red color indicates positive control, light green indicates negative control. Expression of the examined factors was determined in accordance with the scale given in the range from 0 to 100% of the positive control signal (positive control was set as 100%). The bright red color indicates cytokine whose production exceeds the measurement capacity of the method. Explanation of abbreviations of detected factors was placed in supplementary data as Supplementary File F

To examine which factors produced by HEPC-CB.1 cells may be responsible for the observed proangiogenic effects on vascular formation, the profile of cytokine secreted by HEPC-CB.1 cells was evaluated. For screening the HEPC-CB.1 secretome, commercially available C-Series Human Cytokine Antibody Array C1000 was used (Fig. 8). Among the 120 cytokines tested, 63 were detected. Among them were those with known direct pro-angiogenic activities, e.g. VEGF, angiogenin, IL-8, GRO. Moreover, several factors influencing angiogenesis indirectly or modulating this process were detected, e.g. MCP-1, MIP1β, BMP-4, MCSF, SCF, oncostatin and IL-11.

**Fig. 9:**
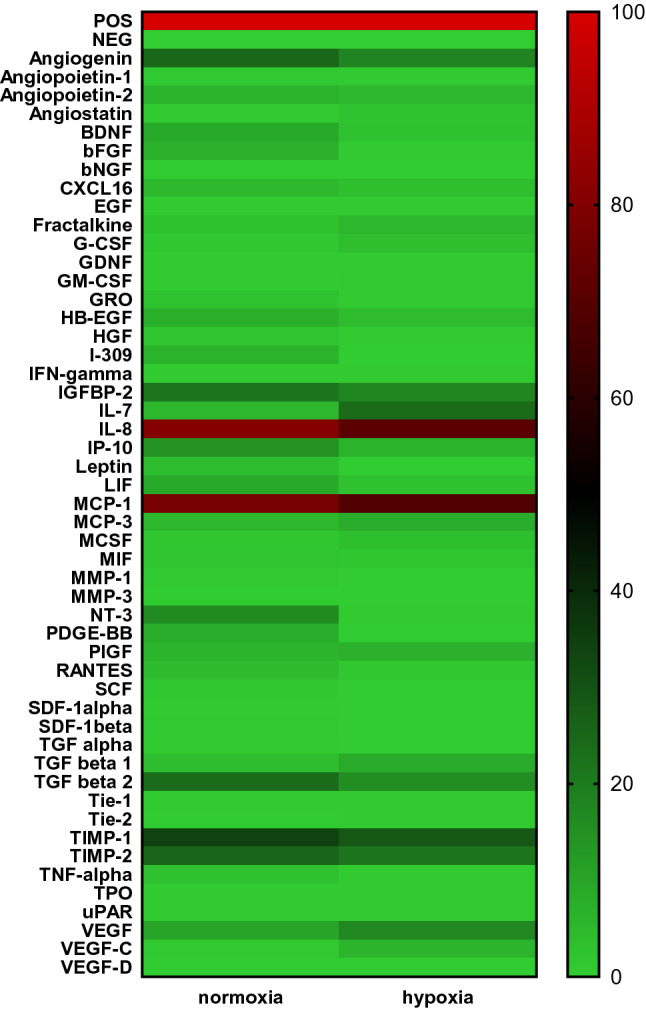
Influence of hypoxia on the production of pro-angiogenic and anti-angiogenic cytokines. Supernatants from the HEPC-CB.1 cell line were collected after 24 h of culture either in normoxia (20% O_2_) or in hypoxia (1% O_2_). Cytokine secretion was evaluated with RayBio Custom C-Series Human Cytokine Antibody Array. Figure presents heatmap of tested cytokines in normoxia versus hypoxia. At the top, dark red color indicates positive control, light green indicates negative control. Expression of the examined factors was determined in accordance with the scale given in the range from 0 to 100% of the positive control signal (positive control set as 100%). Explanation of abbreviations of detected factors was placed in supplementary data as Supplementary File F

To assess the effect of reduced oxygen concentration on the production of factors regulating the process of angiogenesis, the presence of 50 selected factors was assessed using custom antibody array, prepared for individual orders. Under normoxic conditions cells produced 20 and under hypoxic conditions 15 factors (Fig. 9). Among them are factors (1) with known proangiogenic activity, e.g. angiogenin, IL-7, IL-8, MCP-1, MCP-3, VEGF (VEGF-A), VEGF-C, and IGFBP-2, (2) factors whose function depends on the activation of other stimulants, e.g. TGFB1, TGFB2; and (3) anti-angiogenic factors, e.g. IP-10, TIMP-1 and TIMP-2. No major differences were found in normoxic versus hypoxic conditions. Hypoxia generally decreased the expression of secreted factors, although an increase in the expression of proangiogenic cytokine such as VEGF could be noted. Increased IL-7 and VEGF-C secretion in hypoxia was also observed.

## Discussion

The HEPC-CB.1 cell line was obtained from umbilical cord blood [19] and prepared mainly for in vitro and in vivo studies. A similar line of endothelial cell progenitors was presented in Sobhan's paper with the suggestion of using it in tissue engineering [20]. The HEPC-CB.1 cell line has features suggesting that it could be used in some new therapeutic strategies. First of all, HEPC-CB.1 cells are capable of partial differentiation; secondly, these cells are able to integrate and cooperate with the differentiated endothelial cells, improving their tube formation capacity. Moreover, they produce several proangiogenic factors that support angiogenesis and do not express HLA-DR antigen, making possible their safe allogenic application.

Several attempts to differentiate the HEPC-CB.1 line have been made and only partial differentiation of immortalized HEPC-CB.1 cells was achieved with a mixture of ATRA + cAMP + VEGF compounds. The ability of the ATRA + cAMP mixture to differentiate EPC into endothelial cells has been demonstrated so far in mouse embryonic EPC [23]. Demonstration of the role of ATRA and cAMP in the differentiation of other types of cells as well as the process of vascular formation appears in other publications [24–27]. Lack of CD31 protein on the cell surface suggests a block in terminal differentiation of the HEPC-CB.1 cell line. Moreover, cell culture under differentiating conditions also influenced the number of cells capable of colony formation under low-density cell growth. As presented in Fig. 3, after differentiation the percentage of colony forming cells decreases. The morphology of the colony is also changing. After differentiation, numerous colonies with a loose structure appear, which may suggest altered adhesive properties of these cells and/or increased motility. The appearance of the CD34 molecule was also expected. It is postulated that the CD34 molecule appears during differentiation later than CD133, and the expression sequence is as follows: CD133^+^/CD34^−^ phenotype is characteristic for cells that are at the earliest stage of differentiation; cells at the next stage are CD133^+^/CD34^low^ and finally CD133^−^/CD34^+^ [28]. Lack of CD34 expression in HEPC-CB.1 cells, as well as incomplete differentiation, may be the result of the immortalization with a retroviral vector containing the human hTERT telomerase gene. There is some evidence in the literature indicating that immortalized cells lose their ability to differentiate. Only one out of three cell lines obtained from periodontium by transduction with the hTERT gene was successfully differentiated [29]. Despite the incomplete differentiation of HEPC-CB.1 cells, they may be considered as “real” EPC. This statement is additionally supported by the lack of the CD45 antigen. The presence or absence of the CD45 marker is a good distinguishing criterion between “putative” and “real” EPC [30]. Furthermore, HEPC-CB.1 cells do not express either CD117 or CD38, or other molecules typical for hematopoietic cells, and display no sign of differentiation toward erythrocytes, granulocytes or monocytes when cultured in the semisolid differentiation medium MethoCult H4434, which suggests that these cells are not of hematopoietic origin as was presented in our previous publication [19].

So as to distinguish EPC from other cells, including hematopoietic cells, an additional criterion besides the cell phenotype, namely the ability to form capillary-like structures on Matrigel [31], is often used. HEPC-CB.1 cells have the ability to form capillary-like structures. Capillary-like structure formation by HEPC-CB.1 cells is different from and less effective than the mature EC. However, differentiation by the ATRA + cAMP + VEGF mixture was sufficient to increase the efficiency of capillary-like structure formation—differentiated HEPC-CB.1 cells formed 70% more nodes than control cells, and the increase was also observed for two other parameters: mean mesh size and total length.

The results obtained from angiogenic experiments with HEPC-CB.1 and HSkMEC.2 cells labeled with fluorescent dyes indicate that the cells cooperate efficiently in the process of capillary-like structure formation by building a common network. Thus, HEPC-CB.1 cells have a key angiogenic property and therefore fulfill another criterion of real EPC [32, 33]. Moreover, the co-cultures of HEPC-CB.1 with HSkMEC.2 cells form networks with larger meshes, both under normoxic and hypoxic conditions, and a synergic effect can be observed. Also differentiated HEPC-CB.1 cells, similarly to control cells, are able to build into the vascular network formed by HSkMEC.2 and increase the efficiency of network formation as well as undifferentiated cells (data not shown).

It was shown that endothelial progenitor cells exert a proangiogenic effect not only by direct cell–cell contact, but also by the production of active factors and therefore, at least, a part of their activity in angiogenesis is paracrine stimulation of mature endothelial cells [34–36]. It was observed that HSkMEC.2 cells formed networks with a higher number of nodes and longer capillary-like structures in the presence of supernatants from HEPC-CB.1, cultured in both normoxic and in hypoxic conditions. Notably, there were no statistically significant differences in the effects of supernatants collected from normoxia and hypoxia treated cells. These results correlate with the results obtained from cytokine protein array analysis, which indicate that the reduced oxygen concentration did not change significantly the secretion profile of HEPC-CB.1 cells; however, hypoxia induced VEGF C production and augmented production of VEGF. We assume that the proangiogenic effect of HEPC-CB.1 supernatants may be mainly due to the presence of VEGF [37]. Other proangiogenic factors found in supernatants include angiogenin, MIF, IGFBP-2, oncostatin M, MIP 1β, BMP-4, MCSF, NT-3 and SCF [38–45]. However, it should be mentioned that HEPC-CB.1 cells also produce inhibitors of angiogenesis, such as IGFBP-6 [46] and CNTF [47]; therefore the final effect of the supernatant is the result of interplay of pro- and antiangiogenic factors.

It was shown that EPC cells in vivo tend to localize at the site of the fracture of the bone and induce revascularization, which significantly contributed to the repair of bone fractures [48]. This is probably due to the fact that EPC secrete bone morphogenetic proteins. HEPC-CB.1 cells secrete significant amounts of BMP-4 [41], TGF-β1 and IL-11 [49]. This seems particularly important for the therapeutic improvement of difficult bone fractures and indicates a place for potential use of HEPC-CB.1 cells also in orthopedics.

Efforts to use EPC in experimental therapy are so far still not satisfactory and work on angiogenic therapies is still progressing. The success of angiogenic therapy depends on the understanding of EPC biology, which in turn can be explored through research on the established cell lines. The HEPC-CB.1 cell line produces factors that improve the efficiency of capillary-like structure formation by EC cells as well as integrating into the network created by endothelial cells and differentiating toward EC. Moreover, they do not express the antigen HLA-DR, which is responsible for graft rejection. We have demonstrated growth and survival of HEPC-CB.1 cells on vascular biomaterials [50]. All these features indicate that HEPC-CB.1 cells correspond to early progenitors, provide a good cellular model system and an attractive tool for studying the biology of EPC cells and cell-biomaterial interactions, and, finally, could be useful in regenerative medicine.

## Electronic supplementary material

Below is the link to the electronic supplementary material.

Supplementary file1 (DOCX 67 kb)

Supplementary file2 (DOCX 46 kb)

Supplementary file3 (DOCX 351 kb)

Supplementary file4 (DOCX 573 kb)

Supplementary file5 (DOCX 29 kb)
