# Supplementary material for: Activity of the human immortalized endothelial progenitor cell line HEPC-CB.1 supporting in vitro angiogenesis
Source: Mol Biol Rep. 2020 Jul 23;47(8):5911–25. doi: 10.1007/s11033-020-05662-6 (PMC7455590; doi:10.1007/s11033-020-05662-6)
Supplement: Supplementary file 1 — Supplementary file1 (DOCX 67 kb) [file 11033_2020_5662_MOESM1_ESM.docx]

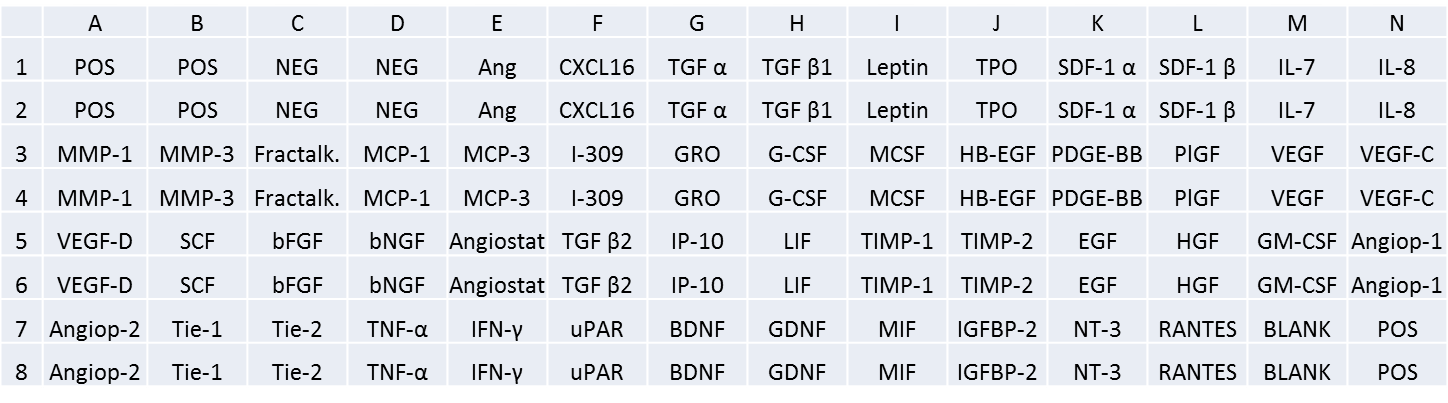


**Supplementary Fig. A.** Map of the RayBio Custom C-Series Human Cytokine Antibody Array protein matrix (RayBiotech Inc.) used in the experiments. POS – positive control test, NEG – negative control test, BLANK – empty field
