# Supplementary material for: Activity of the human immortalized endothelial progenitor cell line HEPC-CB.1 supporting in vitro angiogenesis
Source: Mol Biol Rep. 2020 Jul 23;47(8):5911–25. doi: 10.1007/s11033-020-05662-6 (PMC7455590; doi:10.1007/s11033-020-05662-6)
Supplement: Supplementary file 2 — Supplementary file2 (DOCX 46 kb) [file 11033_2020_5662_MOESM2_ESM.docx]

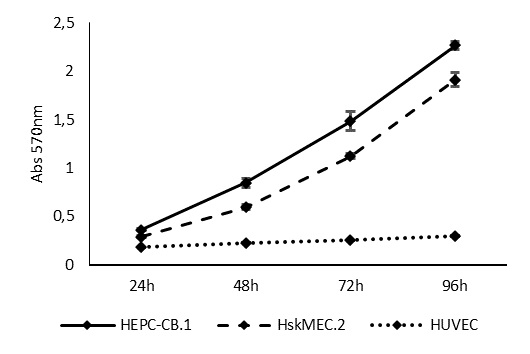


*

*

*

*

*

*

**Supplementary Fig. B.** Comparison the proliferation rate of HEPC-CB.1 cells with EC lines: HSkMEC.2 and HUVEC. The results were obtained using sulforhodamine B tests. The values in the graphs represent the mean absorbance values at 570 nm at time point: 24, 48, 72 and 96h ±SD of the representative experiment determined on the basis of five independent measurements. P values were determined by Student's t test. *Indicates statistically significant differences (p<0.05) between HEPC-CB.1 cells as compared to HSkMEC.2 and HUVEC cells

**Supplementary Fig. C.** Impact of the differentiation process on the proliferation rate of HEPC-CB.1 cell. The results were obtained using sulforhodamine B tests. The values in the graphs represent the mean absorbance values at 570 nm at time point: 24, 48, 72 and 96h ±SD of the representative experiment, determined on the basis of five independent measurements. P values were determined by Student's t test. *Indicates statistically significant differences (p<0.05) between differentiated HEPC-CB.1 cells as compared to HEPC-CB.1 control cells
