# Supplementary material for: Activity of the human immortalized endothelial progenitor cell line HEPC-CB.1 supporting in vitro angiogenesis
Source: Mol Biol Rep. 2020 Jul 23;47(8):5911–25. doi: 10.1007/s11033-020-05662-6 (PMC7455590; doi:10.1007/s11033-020-05662-6)
Supplement: Supplementary file 3 — Supplementary file3 (DOCX 351 kb) [file 11033_2020_5662_MOESM3_ESM.docx]

| **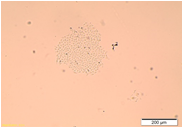**  **200 µm** | **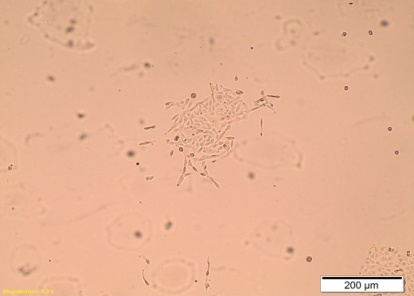**  **200 µm** |
| --- | --- |
| **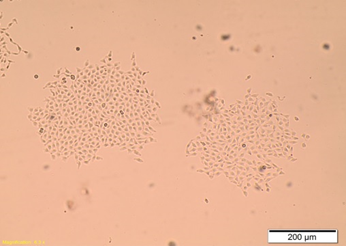**  **200 µm** | **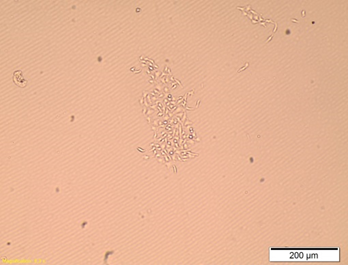**  **200 µm** |
| HEPC-CB.1 control | HEPC-CB.1 differentiated |

**Supplementary Fig. D.** Differentiation process leads to decrease in the cell’s potential to colony formation. The images show the colony (<40 cells) formation by HEPC-CB.1 control and HEPC-CB.1 differentiated cells (magnification 40x). The pictures come from the 6^th^ day of experiment
