# Supplementary material for: Activity of the human immortalized endothelial progenitor cell line HEPC-CB.1 supporting in vitro angiogenesis
Source: Mol Biol Rep. 2020 Jul 23;47(8):5911–25. doi: 10.1007/s11033-020-05662-6 (PMC7455590; doi:10.1007/s11033-020-05662-6)
Supplement: Supplementary file 4 — Supplementary file4 (DOCX 573 kb) [file 11033_2020_5662_MOESM4_ESM.docx]

|  | Normoxia | Hypoxia |
| --- | --- | --- |
| HEPC-CB.1 | 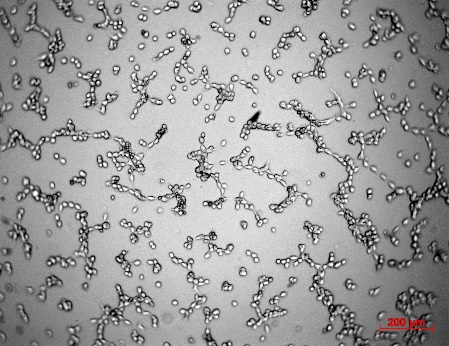  **200 µm** | 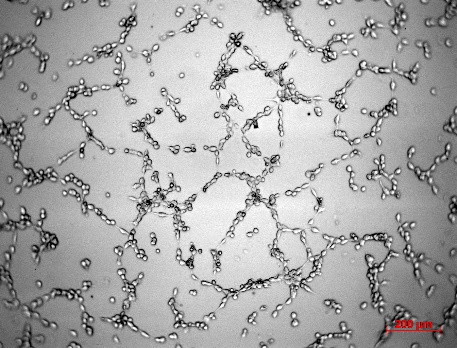  **200 µm** |
| HSkMEC.2 +  HEPC-CB.1 | 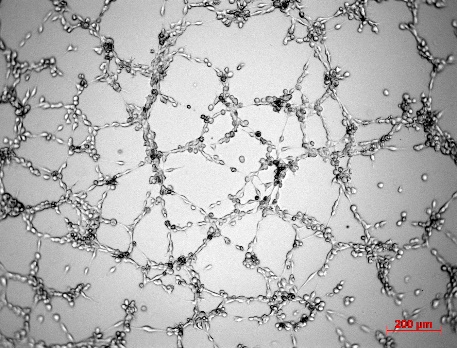  **200 µm** | 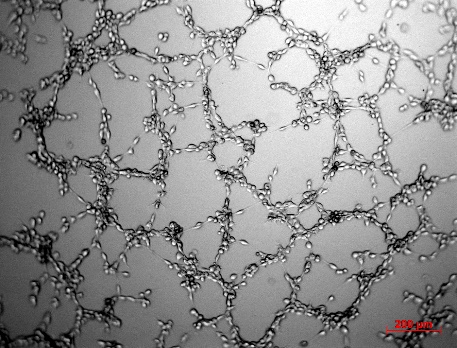  **200 µm** |
| HSkMEC.2 | 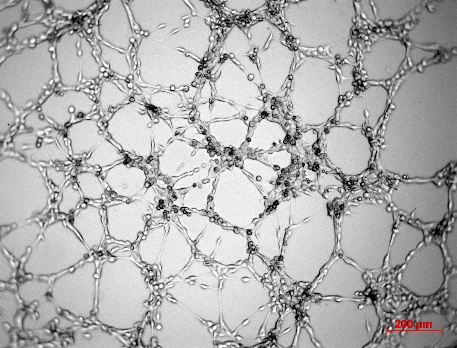  **200 µm** | 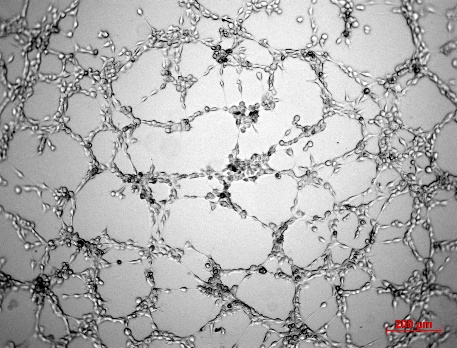  **200 µm** |

**Supplementary Fig. E.** Co-culture of HEPC-CB.1 and HSkMEC.2 cells on Matrigel matrix leads to an increase in the mean mesh size compared to the monoculture of these cells, under both normoxic and hypoxic conditions. Images taken after 18 h of culture using the microscope Axiovert 200M.
