# Supplementary material for: Activity of the human immortalized endothelial progenitor cell line HEPC-CB.1 supporting in vitro angiogenesis
Source: Mol Biol Rep. 2020 Jul 23;47(8):5911–25. doi: 10.1007/s11033-020-05662-6 (PMC7455590; doi:10.1007/s11033-020-05662-6)
Supplement: Supplementary file 5 — Supplementary file5 (DOCX 29 kb) [file 11033_2020_5662_MOESM5_ESM.docx]

Description of the molecules and their full name listed in **Fig. 8:**

**RayBio C-Series Human Cytokine Antibody Array C1000. CytokineAntibody Array part C6:**

| POS | positive control |
| --- | --- |
| NEG | negative control |
| Angiogenin | angiogenin |
| BDNF | brain-derived neurotrophic factor |
| BLC | B lymphocyte chemoattractant, CXCL13 |
| BMP-4 | bone morphogenetic protein 4 |
| BMP-6 | bone morphogenetic protein 6 |
| Ck beta 8-1 | C6 beta-chemokine; CCL23 |
| CNTF | ciliary neurotrophic factor |
| EGF | epidermal growth factor |
| Eotaxin | eotaxin-1; CCL11 |
| Eotaxin-2 | eotaxin-2; CCL24 |
| Eotaxin-3 | eotaxin-3; CCL26 |
| FGF-6 | fibroblast growth factor 6 |
| FGF-7 | fibroblast growth factor 7 |
| Flt-3 Ligand | Fms-related tyrosine kinase 3 ligand |
| Fractalkine | Fractalkine; CX3CL1 |
| GCP-2 | granulocyte chemotactic protein 2; CXCL6 |
| GDNF | glial cell derived neurotrophic factor |
| GM-CSF | granulocyte-macrophage colony-stimulating factor |
| I-309 | T lymphocyte-secreted protein I-309; CCL1; TCA-3 |
| IFN-gamma | interferon gamma |
| IGFBP-1 | insulin-like growth factor-binding protein 2 |
| IGFBP-2 | insulin-like growth factor-binding protein 2 |
| IGFBP-4 | insulin-like growth factor-binding protein 4 |
| IGF-1 | insulin-like growth factor 1 |
| IL-10 | interleukin 10 |
| IL-13 | interleukin 13 |
| IL-15 | interleukin 15 |
| IL-16 | interleukin 16 |
| IL-1 alpha | interleukin 1 alpha |
| IL-1 beta | interleukin 1 beta |
| IL-1 ra | interleukin-1 receptor antagonist |
| IL-2 | interleukin 2 |
| IL-3 | interleukin 3 |
| IL-4 | interleukin 4 |
| IL-5 | interleukin 5 |
| IL-6 | interleukin 6 |
| IL-7 | interleukin 7 |
| Leptin | leptin |
| LIGHT | homologous to lymphotoxin, exhibits inducible expression and competes with HSV glycoprotein D for binding to herpesvirus entry mediator, a receptor expressed on T lymphocytes; TNFSF14 |
| MCP-1 | monocyte chemoattractant protein-1; CCL2 |
| MCP-2 | monocyte chemoattractant protein-2; CCL8 |
| MCP-3 | monocyte chemoattractant protein-3; CCL7 |
| MCP-4 | monocyte chemoattractant protein-4; CCL13 |
| M-CSF | macrophage colony-stimulating factor |
| MDC | macrophage-derived chemokine; CCL22 |
| MIG | monokine induced by gamma-INF; CXCL9 |
| MIP-1 delta | macrophage inflammatory proteins 1 delta; CCL15 |
| MIP-3 alpha | macrophage inflammatory proteins 3 alpha; CCL20 |
| NAP-2 | neutrophil activating peptide 2; CXCL7 |
| NT-3 | neurotrophin-3 |
| PARC | p53-associated parkin-like cytoplasmic protein; CCL18 |
| PDGF-BB | platelet-derived growth factor-BB |
| RANTES | regulated upon activation, normal T cell expressed and secreted; CCL5 |
| SCF | stem cell factor |
| SDF-1 | stromal cell-derived factor alpha 1; CXCL12 alpha |
| TARC | thymus and activation-regulated chemokine; CCL17 |
| TGF beta 1 | transforming growth factor beta 1 |
| TGF beta 3 | transforming growth factor beta 3 |
| TNF alpha | tumor necrosis factor alpha |
| TNF beta | tumor necrosis factor beta |

**RayBio C-Series Human Cytokine Antibody Array C1000. CytokineAntibody Array part C7:**

| POS | positive control |
| --- | --- |
| NEG | negative control |
| Acrp30 | adiponectin |
| AgRP | agouti-related peptide |
| Angiopoietin-2 | angiopoietin-2 |
| Amphiregulin | amphiregulin |
| Axl | AXL receptor tyrosine kinase; ARK |
| bFGF | basic fibroblast growth factor |
| beta-NGF | beta-nerve growth factor |
| BTC | betacellulin |
| CCL28 | C-C Motif Chemokine Ligand 28 |
| CTACK | cutaneous T-cell-attracting chemokine; CCL27 |
| Dtk | Tyrosine-protein kinase DTK |
| EGF-R | epidermal growth factor receptor |
| ENA-78 | epithelial-neutrophil activating peptide, CXCL5 |
| Fas/TNFRSF6 | tumor necrosis factor receptor superfamily member 6; APO-1; CD95 |
| FGF-4 | gibroblast growth factor 4 |
| FGF-9 | fibroblast growth factor 9 |
| G-CSF | granulocyte colony-stimulating factor |
| GITR Ligand | glucocorticoid-induced TNF-related ligand; TNFSF18 |
| GITR | glucocorticoid-induced TNFR-related protein; TNFRSF18 |
| GRO | growth related oncogene alpha/beta/gamma |
| GRO alpha | growth related oncogene alpha; CXCL1 |
| HCC-4 | chemokine CC-4; CCL-16 |
| HGF | hepatocyte growth factor |
| ICAM-1 | intercellular adhesion molecule 1, CD54 |
| ICAM-3 | intercellular adhesion molecule 3, CD50 |
| IGF-BP-3 | insulin-like growth factor-binding protein 3 |
| IGF-BP-6 | insulin-like growth factor-binding protein 6 |
| IGF-1 SR | insulin-like growth factor 1 receptor |
| IL-1 R4/ST2 | interleukin 1 receptor 4; ST2 |
| IL-1 R1 | interleukin 1 receptor 1 |
| IL-11 | interleukin 11 |
| IL-12 p40 | interleukin-12 subunit p40 |
| IL-12 p70 | interleukin-12 subunit p70 |
| IL-17 | interleukin 17 |
| IL-2 Ra | interleukin 2 receptor alpha |
| IL-6 R | interleukin 6 receptor |
| IL-8 | interleukin 8; CXCL8 |
| I-TAC | interferon-inducible T-cell alpha chemoattractant; CXCL 11 |
| Lymphotactin | lymphotactin; XCL1 |
| MIF | macrophage migration inhibitory factor |
| MIP-1 alpha | macrophage inflammatory protein-1 alpha; CCL3 |
| MIP-1 beta | macrophage inflammatory protein-1 beta; CCL4 |
| MIP-3 beta | macrophage inflammatory protein-3 beta; CCL19 |
| MSP a | macrophage-stimulating protein alpha/beta |
| NT-4 | neurotrophin-4 |
| Osteoprotegerin | osteoprotegerin; TNFRSF11B |
| Oncostatin M | oncostatin M |
| PLGF | placenta growth factor |
| sgp130 | glycoprotein 130 |
| sTNF RII | soluble tumor necrosis factor receptor II |
| sTNF RI | soluble tumor necrosis factor receptor I |
| TECK | thymus-expressed chemokine; CCL25 |
| TIMP-1 | tissue inhibitor of metalloproteinases 1 |
| TIMP-2 | tissue inhibitor of metalloproteinases 2 |
| TPO | thrombopoietin |
| TRAIL R3 | tumor necrosis factor-related apoptosis-inducing ligand receptor 3 |
| TRAIL R4 | tumor necrosis factor-related apoptosis-inducing ligand receptor 4 |
| uPAR | urokinase-type plasminogen activator receptor |
| VEGF | vascular endothelial growth factor A |
| VEGF-D | vascular endothelial growth factor D |

Description of the molecules and their full name listed in **Fig. 9. RayBio Custom C-Series Human Cytokine Antibody Array:**

| POS | positive control |
| --- | --- |
| NEG | negative control |
| Angiogenin | Angiogenin |
| Angiopoietin-1 | Angiopoietin-1 |
| Angiopoietin-2 | Angiopoietin-2 |
| Angiostatin | Angiostatin |
| BDNF | brain-derived neurotrophic factor |
| bFGF | basic fibroblast growth factor |
| bNGF | beta nerve growth factor |
| CXCL16 | C-X-C motif chemokine ligand 16 |
| EGF | epidermal growth factor |
| Fractalkine | Fractalkine; CX3CL1 |
| G-CSF | granulocyte colony-stimulating factor |
| GDNF | glial cell derived neurotrophic factor |
| GM-CSF | granulocyte-macrophage colony-stimulating factor |
| GRO | growth related oncogene |
| HB-EGF | heparin binding EGF like growth factor |
| HGF | hepatocyte growth factor |
| I-309 | T lymphocyte-secreted protein I-309; CCL1; TCA-3 |
| IFN-gamma | interferon gamma |
| IGFBP-2 | insulin-like growth factor-binding protein 2 |
| IL-7 | interleukin 7 |
| IL-8 | interleukin 8 |
| IP-10 | interleukin 10 |
| Leptin | leptin |
| LIF | leukemia inhibitory factor |
| MCP-1 | monocyte chemoattractant protein-1; CCL2 |
| MCP-3 | monocyte chemoattractant protein-3; CCL7 |
| MCSF | macrophage colony-stimulating factor |
| MIF | macrophage migration inhibitory factor |
| MMP-1 | matrix metalloproteinase-1 |
| MMP-3 | matrix metalloproteinase-1 |
| NT-3 | neurotrophin-3 |
| PDGE-BB | platelet-derived growth factor-BB |
| PlGF | placenta growth factor |
| RANTES | regulated upon activation, normal T cell expressed and secreted; CCL5 |
| SCF | stem cell factor |
| SDF-1alpha | stromal cell-derived factor alpha 1; CXCL12 alpha |
| SDF-1beta | stromal cell-derived factor beta 1; CXCL12 beta |
| TGF alpha | transforming growth factor alpha |
| TGF beta 1 | transforming growth factor beta 1 |
| TGF beta 2 | transforming growth factor beta 2 |
| Tie-1 | tyrosine kinase with immunoglobulin-like and EGF-like domains 1 |
| Tie-2 | tyrosine kinase with immunoglobulin-like and EGF-like domains 2; CD202b |
| TIMP-1 | tissue inhibitor of metalloproteinases 1 |
| TIMP-2 | tissue inhibitor of metalloproteinases 2 |
| TNF-alpha | tumor necrosis factor alpha |
| TPO | thrombopoietin |
| uPAR | urokinase-type plasminogen activator receptor |
| VEGF | vascular endothelial growth factor A |
| VEGF-C | vascular endothelial growth factor C |
| VEGF-D | vascular endothelial growth factor D |
